# Is telemonitoring an option against shortage of physicians in rural regions? attitude towards telemedical devices in the North Rhine-Westphalian health survey, Germany

**DOI:** 10.1186/1472-6963-12-95

**Published:** 2012-04-16

**Authors:** Claudia Terschüren, Monika Mensing, Odile CL Mekel

**Affiliations:** 1 NRW Centre for Health, Westerfeldstr. 35-37, Bielefeld 33611, Germany

**Keywords:** Attitude, Home telemonitoring, Germany, Population-based survey, Telemedical devices, Telephone survey

## Abstract

**Background:**

General practitioners (GP) in rural areas of Germany are struggling to find successors for their private practices. Telemonitoring at home offers an option to support remaining GPs and specialists in ambulatory care.

**Methods:**

We assessed the knowledge and attitude towards telemedicine in the population of North Rhine-Westphalia (NRW), Germany, in a population-based telephone survey.

**Results:**

Out of 2,006 participants, 734 (36.6%) reported an awareness of telemedical devices. Only 37 participants (1.8%) have experience in using them. The majority of participants were in favour of using them in case of illness (72.2%). However, this approval declined with age. These findings were similar in rural and urban areas. Participants who were in favour of telemedicine (n = 1,480) strongly agreed that they would have to see their doctor less often, and that the doctor would recognize earlier relevant changes in their vital status. Participants who disliked to be monitored by telemedical devices preferred to receive immediate feedback from their physician. Especially, the elderly fear the loss of personal contact with their physician. They need the direct patient-physician communication.

**Conclusions:**

The fear of being left alone with the technique needs to be compensated for today's elderly patients to enhance acceptance of home telemonitoring as support for remaining doctors either in the rural areas or cities.

## Background

In rural regions of Germany, a shortage of physicians exists. Especially, general practitioners (GPs) are struggling to find successors [1]. Up to 15,000 general practitioners would be needed to guarantee today's level of ambulatory care by GPs (n = 60,374) in 2020-2025 [2]. Also in North Rhine-Westphalia (NRW), Germany, there are rural regions where GPs are retiring without finding a successor. Since physicians' practices better persist in urban areas, distances to see a doctor are increasing. Waiting lists for appointments are extending. These challenges caused concern among patients and mayors of remote towns or villages. Citizens started to sign petitions for the preservation of the ambulatory practices in their municipalities [3]. They posted calls via Internet [4] and television [5] to find a new doctor for their community.

Experts noted a reluctance among German medical students and young physicians to work in primary care or hospitals [6,7]. Financial incentives for GPs in remote areas [2], recruitment of medical students from rural origin [8], specific funding for advanced training of GPs [1] and extended possibilities for delegation by the GP to specifically trained assistants [9,10] were given as suggestions to counteract the predicted increase in physician shortage.

Home telecare and telemonitoring are prospective options to support remaining GPs and specialists' ambulatory care practices in less populated areas. Several reviews provide first clinical evidence for the benefit of telemedicine in specific patient groups, especially those with chronic diseases [11-14]. However, in Germany, people are not used to conditions like in other European countries, e.g. in northern Sweden or Norway where in remote sparsely populated regions telemonitoring networks are already established.

Until now, it is rather unclear whether or not the general population in Germany is ready for the implementation of telemonitoring as part of their regional ambulatory care system. The existence of positive attitudes towards the use of telemedicine technologies is one important prerequisite for a successful implementation in primary health care. In NRW, a predominantly rural region (East Westphalia-Lippe) was elected in 2011 as exemplary model region for comprehensive use of telemedicine in health care [15].

We investigated the population's awareness of home telemonitoring in health care, and its attitude towards the use of telemedical devices. We also analysed if the rural population already might differ in acceptance of the telemedicine option. Results of the survey reflect the base line before projects would be conducted within the model region and are supposed as information for physicians, politicians and further involved stakeholders.

## Methods

To evaluate knowledge and attitude towards telemedical devices in the population of NRW, Germany, we integrated a specific module in the 6th telephone survey conducted by the NRW Centre for Health. It was funded by the Ministry of Work, Health, and Social Affairs of NRW.

The NRW health survey is conducted on a regular yearly basis to assess and document the health status of the NRW population. It serves as a tool to identify health needs, initiate policies, and to evaluate effects on population health. Selection criteria include residence in the federal state of NRW, 18 years of age or older, and a telephone mainline as part of the fixed line telephone network.

A two-stage sampling was conducted. The first stage of sampling based on the random digit dialling method of Gabler and Häder [16] to select the participating households. The second stage of sampling involved random selection of the target person within the household members using the last-birthday method [17,18].

An opinion research institute (Sozialwissenschaftliches Umfragezentrum GmbH (SUZ), Duisburg, Germany) carried out the interviews from November 18, 2009 to December 14, 2009, on the basis of a questionnaire developed at the NRW Centre for Health. Potential participants were contacted on weekdays from 4:30 pm to 9:00 pm and on Saturday from 12:00 pm to 6:00 pm to guarantee the inclusion of the working population.

**Figure 1:**
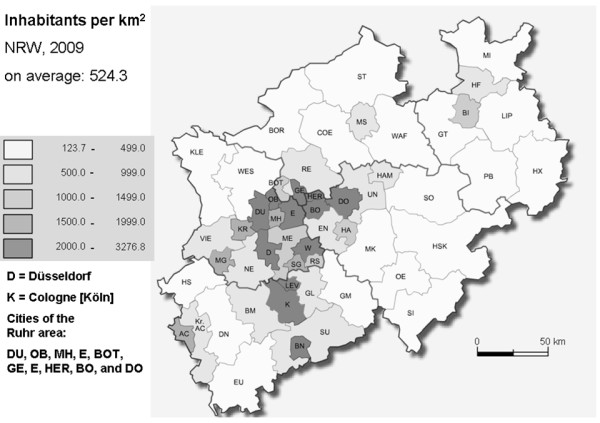
**Distribution of the population density in NRW**.

NRW has 18 million inhabitants and is the most populated federal state of Germany. The federal state includes densely populated cities like Düsseldorf (capital city, 2,698.8 inhabitants per km^2^), or Cologne (2,463.5 inhabitants per km^2^), and the Ruhr area which comprises 11 of these densely populated cities (> 3,000 inhabitants/km^2^: n = 1, > 2,000-3,000 inhabitants/km^2^: n = 6, > 1,000-2,000 inhabitants/km^2^: n = 4). On average, NRW has a population density of 524.3 inhabitants/km^2 ^(Figure 1). We categorized participants with residency in municipalities with equal or less than (≤) 500 inhabitants per square kilometres (km^2^) as living rural (urban > 500 inhabitants/km^2^).

Interviews were conducted using the Computer Assisted Telephone Interviewing (CATI) method. The participants were asked for self-rated health status, present diseases (chronic and communicable), health care utilization, and prevention. Socio-demographic parameters, healthy lifestyle, and smoking habits were also ascertained. A pretest was conducted to check for possible ambiguous or unclear questions or wording. According to statutes of the Medical Association North Rhine the responsible ethic committee has to judge ethical and judicial aspects of biomedical research projects with humans and of epidemiological research with individual-related data which include identifying variables. For our telephone survey we ascertained anonymous information only, so we did not qualify for an application. The survey was not approved by the ethics committee. Therefore, survey questions were diligently discussed in house.

Each participant was informed a priori about the purpose and content of the survey. The interviewer had to explain, that the potential participant has the right to refuse to take part, or to stop at any question, and that data will be anonymous.

The questionnaire consisted of 110 questions in total. Our specific telemedicine module included 11 questions on awareness of telemedical devices used to monitor diseases at home and attitude towards the individual use of telemedicine in case of illness. These questions were developed for this survey. The module included a short description of the function and purpose of telemedical devices to ensure a consistent definition for each participant which was read out by the interviewer. Telemedical devices were explained as measurement equipment which allows patients to transfer data of e.g. weight, blood pressure, or an electrocardiogram via telephone line or Internet from patients' homes to the physicians practice. The technique is most often used to closely monitor patients with heart disease or diabetes from distance in a short interval of time.

Questions were tailored to adapt the preceding information based on a person's awareness of this application. To ascertain the attitude towards the use of telemedical devices theses on possible advantages and disadvantages were read out to the participants. Additionally, participants could report their own theses (optional free text). The participants chose from the following categories for their estimation: "completely true", "true", "half/half", "less true", "not true", "don't know", and "not applicable". Participants in favour of using telemedicine at home were asked how much they would agree with the following statements associated with this technique. Given theses for estimation were:

- "Due to the frequent data transfer of measured values, my doctor will realize earlier a decrease of my health status" (abbreviated as: early recognition)

- "Visits at the doctor's office will be less often." (less visits)

- "I myself will be able to control if my health status is good or increasing." (self monitoring)

- "Measuring and transferring the data to my doctor everyday motivates me to follow a healthier life style." (incentive)

- Further advantages (optional free text)

Participants who disliked the idea of using telemedicine in a case of illness were asked how much they agree with disadvantages that might occur. Theses given were:

- "I feel more confident if the measurements are done by the doctor him-/herself or by a nurse at his/her practice." (abbreviated as: no supervision)

- "I am afraid of additional costs due to the new technique." (additional costs)

- "I want to talk to my doctor personally about the results of the measurement." (no immediate feedback)

- "I have doubts because of data safety and protection." (data protection)

- Other reasons (optional free text)

If participants reported to be aware of telemedical devices, they were asked about the source of information in a multi-response question (e.g. physician, friends, television, Internet).

Proportions were calculated to show the distribution of answer categories and distribution by subgroups. To check for statistically significant differences we used the χ^2^-test. We used SPSS V15 (Statistical Package for Social Sciences Inc., IBM company, Chicago, Illinois, USA) for the analyses.

## Results

### Characteristics of survey population

The response rate was 64.1%. In total, 2.006 persons participated in the telephone survey (995 men; 1011 women). Of these, 439 (21.9%) participants were 65 years of age or older. The age ranged from 18 to 93 years (Table 1).

**Table 1:** Characteristics of the survey population

characteristics	number (n)	SD, range
mean age *	1999	48.8 (17.03, 18-93)

		**in percent (%)**

females	1011	50.4

lives with family/partner	1555	77.8

school leaving certificate - Abitur (German:qualification for university)	658	32.8

ISCED**		
- low education (ISCED 1-2)	135	6.8
- medium education (ISCED3A, 3B, 4A)	1223	61.2
- high education (ISCED 5,6)	489	24.5
- still student/trainee	149	7.5

migration background	445	22.2

self-rated health status "good" to "very good"	1508	75.2

diagnosed with diabetes	40	2.0

diagnosed with hypertension	512	25.5

diagnosed with both, diabetes and hypertension	86	4.3

* age is unknown for 7 participants, SD = standard deviation** education according to ISCED-1997: International Standard Classification of Education 1997 [19]

### Awareness in the population and sources of information

**Figure 2:**
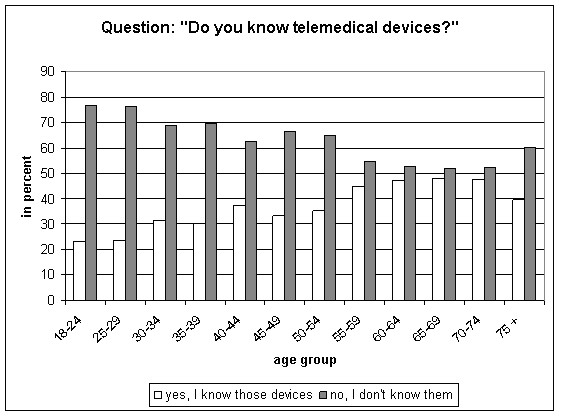
**Awareness of home telemedical devices by age group**.

Out of 2,006 participants, 734 (36.6%) were aware of telemedical devices. More men (41.8%) than women (31.5%) reported to be aware of this technical development in health care. In the participants < 65 years, the proportion of those who stated to know such devices increased by age group. Highest awareness was observed in the age group 65 to 69 years (48.1%; Figure 2). However, only 37 (1.8%) participants were using those devices at the time of the interview or had used them before.

The awareness differed by education with statistical significance. Of those who achieved the qualification to go to university (*Abitur*: 32.8%, Table 1) 40.2% reported having knowledge of telemedical devices, whereas in participants with less than high school graduation only 34.6% know of these devices (p = 0.019).

Those who reported to be aware of home telemedicine (n = 734) most often read a respective article in a magazine (51.4%), or watched television programs referring to this topic (48.8%). The Internet was named by 22.9% of the participants as source of information, 25.1% reported family members or friends who used telemedical devices. A physician was the source of information for 18.0% (n = 132). At least 12.3% of all participants learned about telemedicine at their work place. In the age group 18-49 years, 18.4% of the interviewees reported the work place as source.

Participants 65 years of age or older who stated to know telemedical devices (n = 199 of 439; 45.3%) most often reported that they learned about telemedicine by reading a respective article in a magazine (63.3%), followed by watching television (54.3%). For persons younger than 30 and informed about this technique (n = 72 of 308; 23.4%), main sources were family or friends (40.3%), Internet or magazines (both 36.1%) and the work place (22.2%).

The source of information was influenced by health status. Those participants who had been diagnosed with diabetes and/or hypertension more often reported that their physician told them about telemedical devices (diabetes and/or hypertension: 27.4%, none of these diagnoses: 14.1%; p = 0.002).

### Attitude towards telemedical devices

**Figure 3:**
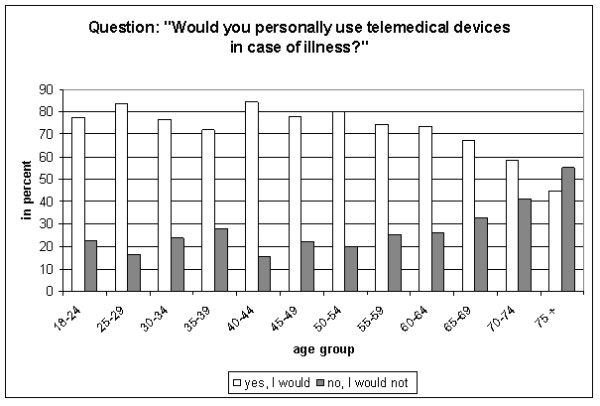
**Approval for use of telemedicine by age group**.

Despite the fact that only 37 participants had used such devices personally, the majority of non-users approved the idea to use them themselves in case of illness (n = 1449; 72.2%). However, almost one fifth disliked the idea (n = 363; 18.1%). Indecisive were 156 participants (7.8%; 1 person without statement). More men (n = 746; 75.4%) than women (n = 703, 70.9%) liked the idea of telemedical support in case of illness (p = 0.005). Approval to use telemedicine personally in case of illness declined in the elderly (> 65 years). Finally, in the age group 75+, more participants refused than approved telemonitoring (Figure 3).

A majority of those participants who reported to use telemedical devices at the time of the interview or had used such devices had a positive attitude towards this technique. In total, 31 (83.8%) of 37 persons approved repeated use of telemonitoring. Four persons in this group (10.8%) would object to use telemedical devices in the future and two were indecisive.

### Approval independent of rurality

In participants living in rural areas, 79.6% approved the personal use of telemedical devices in case of illness. In urban areas, 80.3% would accept such telemonitoring. In total, we found no differences by population density (Table 2). Gender had an impact in rural as well as in urban areas (approval of telemonitoring in case of illness: men, rural: 82.1% vs. men, urban: 83.4%; women, rural: 76.9% vs. women, urban: 77.4%). The decline of approval by age was similar in rural and urban areas. Disapproval was highest in the oldest age group in all four categories. Especially in females 70+ living in urban areas, only 53.5% would use telemedical devices (females, rural: 62.1%; Table 2).

**Table 2:** Use of telemedical devices in case of illness by rural vs. urban residency

males								
**age group**	**rural (≤ 500 inh./km^2^)**				**urban (> 500 inh./km^2^)**			
	
	**positive^1^**		**negative^2^**		**positive^1^**		**negative^2^**	
	
	N	%	n	%	n	%	n	%

18-29	32	76.2	10	23.8	103	82.4	22	17.6

30-39	29	90.6	3	9.4	68	82.9	14	17.0

40-49	70	92.1	6	7.9	117	88.0	16	12.1

50-59	39	86.7	6	13.3	110	87.3	16	12.7

60-69	28	70.0	12	30.0	86	85.1	15	14.8

70 +	22	66.7	11	33.4	57	69.5	25	30.5

total	220	82.1	48	17.9	541	83.4	108	16.7
**females**								

**age group**	**rural (≤ 500 inh./km^2^)**				**urban (> 500 inh./km^2^)**			
	
	**positive^1^**		**negative^2^**		**positive^1^**		**negative^2^**	
	
	n	%	n	%	n	%	n	%

18-29	26	89.7	3	10.3	85	90.4	9	9.5

30-39	31	72.1	12	27.9	91	80.5	22	19.5

40-49	51	82.3	11	17.8	136	82.9	28	17.1

50-59	36	78.3	10	21.8	99	79.8	25	20.1

60-69	24	72.7	9	27.3	64	72.7	24	27.3

70 +	18	62.1	11	37.9	53	53.5	46	46.4

total	186	76.9	56	23.1	528	77.4	154	22.6

inh. = inhabitants

^1 ^sum of answers: "yes" and "rather yes"

^2 ^sum of answers: "rather no" and "no"

### Reasons for approval or objection

Participants who were in favour of telemedicine (n = 1,480) strongly agreed with the advantage that telemonitoring would help to reduce visits to their physician's practice (58.4%). In women, even 62.3% agreed to this statement (Table 3 "less visits"). Both, men and women, agreed that their physician would be able to recognize earlier relevant changes in the vital status of the patients due to the continuous data transfer (Table 3 "early recognition"). Of less importance were the options to have better control about the own health status, and to understand the measurement results as an instrument to motivate the person to maintain a certain health status (Table 3"self monitoring" and "incentive").

**Table 3:** Statements supporting the use of telemedical devices

answer categories	early recognition				less visits				self monitoring				incentive			
	**men**		**women**		**men**		**women**		**men**		**women**		**men**		**women**	

	**n**	**%**	**n**	**%**	**n**	**%**	**n**	**%**	**n**	**%**	**n**	**%**	**n**	**%**	**n**	**%**

completely true	430	56.4	391	54.5	417	54.7	447	62.3	255	33.4	267	37.2	189	24.8	187	26.1

true	235	30.8	235	32.8	198	26.0	156	21.8	272	35.6	249	34.7	226	29.6	227	31.7

half/half	24	3.1	22	3.1	24	3.1	16	2.2	34	4.5	44	6.1	52	6.8	39	5.4

less true	46	6.0	37	5.2	73	9.6	66	9.2	137	18.0	112	15.6	215	28.2	184	25.7

not true	6	0.8	11	1,5	35	4.6	25	3.5	40	5.2	31	4.3	63	8.3	62	8.6

don't know	15	2.0	19	2.6	12	1.6	5	0.7	14	1.8	12	1.7	12	1.6	15	2.1

not specified	7	0.9	2	0.3	4	0.5	2	0.3	11	1.4	2	0.3	6	0.8	3	0.4

total*	763	100.0	717	100.0	763	100.0	717	100.0	763	100.0	717	100.0	763	100.0	717	100.0

* 1449 persons who have not used telemedical devices until the interview was conducted, but approved the use, plus 31 persons who already used those devices personally and would do so again in the future (n = 1480)

Participants objecting to home telemonitoring in the case of illness strongly supported two theses given in the questionnaire. The majority opposed the use of telemedicine because they preferred to receive immediate feedback from their physician referring to the new values (Table 4 "no immediate feedback"). Additionally, they would feel more confident if the measurements are conducted by the doctor himself or by a nurse at the physicians' practice (Table 4 "no supervision"). Less important reasons of refusal were additional costs, or possible data loss and other data protection problems (Table 4 "additional costs" and "data protection"). There were no statistically significant differences between men and women.

**Table 4:** Statements opposing the use of telemedical devices

answer categories	no supervision				additional costs				no immediate feedback				data protection			
	**men**		**women**		**men**		**women**		**men**		**women**		**men**		**women**	

	**n**	**%**	**n**	**%**	**n**	**%**	**n**	**%**	**n**	**%**	**n**	**%**	**n**	**%**	**n**	**%**

completely true	80	51.3	98	46.4	22	14.1	28	13.3	105	67.3	131	62.1	37	23.7	41	19.4

true	23	14.7	40	19.0	20	12.8	36	17.1	23	14.7	32	15.2	15	9.6	29	13.7

half/half	5	3.2	16	7.6	9	5.8	13	6.2	7	4.5	7	3.3	11	7.1	13	6.2

less true	22	14.1	24	11.4	47	30.1	64	30.3	8	5.1	19	9.0	41	26.3	59	28.0

not true	18	11.5	20	9.5	51	32.7	63	29.9	8	5.1	13	6.2	45	28.8	62	29.4

don't know	4	2.6	11	5.2	4	2.6	7	3.3	2	1.3	8	3.8	4	2.6	6	2.8

not specified	4	2.6	2	0.9	3	1.9	0	0.0	3	1.9	1	0.5	3	1.9	1	0.5

total*	156	100.0	211	100.0	156	100.0	211	100.0	156	100.0	211	100.0	156	100.0	211	100.0

* 363 persons who have not used telemedical devices until the interview was conducted and disapproved the use plus 4 persons who already used those devices and dislike a repeated use (n = 367)

Positive attitude towards telemedical devices was age-dependent. While in the age group 65 or older (65+), 41.4% agreed completely to the thesis of early recognition, the approval was 58.9% in the youngest group (18-29 years; p < 0.0001). A similar distribution was seen for the advantage of "less visits" (65+: 41.4%; 18-29: 61.4%; p < 0.0001). No difference by age was observed for "self monitoring" (65+: 35.5%; 18-29: 34.1%; p = 0.979) and "incentive" (65+: 24.2%; 18-29: 24.8%; p = 0.820).

In those participants who opposed the idea of telemonitoring we also found an age gradient. More elderly than younger participants were skeptical of telemedical devices. While in the eldest age group (65+), more than two-thirds (68.0%) completely agreed with the thesis that they prefer to talk to their physician personally after the measurement, it was 59.1% in the youngest group (18-29 years). However, after pooling the categories "completely true" and "true" for this thesis the agreement reached about 80% in all age groups (age group 18-29: 81.8%; 30-44: 77.7%; 45-64: 77.1%; 65+: 82.1%).

**Figure 4:**
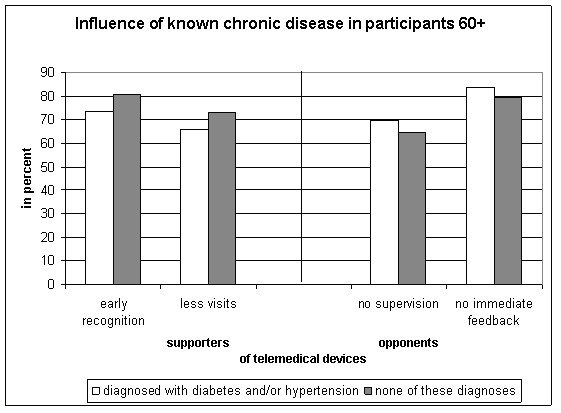
**Influence of known chronic disease on agreement with eligible statements**.

Since older age and chronic diseases are related, answer categories of the theses were stratified by the variables "diagnosed with diabetes, hypertension, or both" vs. "none of these two diseases" for participants 60 years or older. Of those who approved to use telemedical devices in case of illness were 440 (29.7%) participants diagnosed with diabetes and/or hypertension (60+: n = 223). The participants 60+ and diagnosed with diabetes and/or hypertension were less confident than those 60+ without these diagnoses, that telemonitoring helps their physician to identify a decreasing health status earlier (Figure 4). In those who disliked the idea of telemonitoring more participants 60+ with diabetes and/or hypertension than without these diseases wanted the doctor to conduct the measurements and feared to receive no immediate feedback (Figure 4).

## Discussion

In general, the survey showed that telemedical devices are not well known (37%) in the NRW population. Personal experience in use of those devices was reported by only 2%. Even if the majority of our survey participants approved the idea of using the devices in case of illness, this positive attitude was strongly age-dependent. In the elderly (55+), the proportion of participants who disliked the use of telemedical devices was slightly increasing. In the age group 75+ more opponents than supporters of telemonitoring at home existed. The age group in the population who could benefit most due to a high proportion of chronically ill patients showed most often refusal. This finding is comparable to the results of a British study by Mair et al. (2006, [20]). This randomized controlled home telecare trial with predominantly older patients with chronic obstructive pulmonary disease (COPD) in a state of acute exacerbation showed that the likelihood of consent decreased by age (per one year older: OR = 0.96, 95% CI 0.93-0.98, p = 0.001).

The participants in the older age groups were more often aware of the telemedical devices than the younger ones. Older age and prevalence of chronic diseases like diabetes, hypertension, or ischemic heart disease are associated. In Germany, more than 50% are diagnosed with hypertension by their treating physician in the age group 65 or older [21]. The prevalence of diabetes mellitus type 2 in the German population is higher than 20% in the age group 70 or older [22]. As a consequence, the proportion of participants who are affected by a chronic disease is higher among older age groups. Those survey participants who already were diagnosed with a chronic disease were more often informed about telemedical devices by their treating physician. They are potential candidates to benefit from telemedicine treatment [23].

In contrast to age, the location of residency - rural or urban - did not have an influence on the attitude towards the personal use of telemedical devices in our sample. Either rural or urban, the lowest approval of personally using those devices in case of illness was in participants older than 70 years of age. The difference of almost 10% between women 70+ in rural and urban areas might be due to the small sample size in this age group (females, rural, 70+: n = 29 in total). The attitude has been similar so far, even despite the fact that in some villages the citizens already experience the (potential) disappearance of the GP in a favourable proximity.

The IDEATel project showed that primary care providers in underserved areas of upstate New York approved telemedicine because of more patient control and motivation. Having extra patient data was perceived as helpful [24]. These positive aspects are similar to the expectations the participants were voicing in our survey. The majority of participants who are in favour of telemonitoring in the case of illness want their physicians to recognize earlier relevant changes of their vital status (Table 3).

Those participants who disapproved the use of telemedical devices were mainly concerned about receiving no direct feedback from their doctor (Table 4). Mostly, these persons were elderly participants. This finding is comparable to a Danish survey which investigated the attitudes towards telehealth use among residents in a rural area. The survey showed that 58% of the participants disliked the idea of having a video consultation with a specialist doctor [25]. The reluctance against the video consultation was significantly higher among older people (age group 70 to 80 years: OR = 2.69; p < 0.01).

A recent survey of older Hong Kong people's perception of telecare devices [26] showed that the participants (65 years or older) were positive about the function and usefulness of the devices, but also stated they would not use them personally. In our survey, more participants with diabetes and/or hypertension than participants without such diseases were anxious to receive no immediate feedback from their treating physician. In those persons for whom these devices are developed to be helpful the possible impact of less contact with the treating physician enhances concern. A recent German review on home telemonitoring in patients with chronic congestive heart failure found evidence for a positive effect on clinical endpoints, particularly mortality, but concluded that improvement of patient-reported outcomes still needs to be demonstrated [14].

In a pilot project (28 patients) to assess efficiency and experience of teledermatology in primary care in Canada, the patients only preferred teleconsulting for referral when available significantly sooner than face-to-face appointments [27]. A German intervention study comprised the use of telemedical devices and the delegation of home visits to qualified practice assistants [28,29]. In total, 105 patients participated in the project. Out of these, 48 patients used telemedical devices to monitor health parameters. 87.4% of the patients accepted the combination of telecare and qualified practice assistants as comparable to usual care by their GP [29].

The telephone survey used in this study was restricted to persons accessible by landline. However, random sampling, calls in the evening hours, and the fact that 90% of all households in Germany [30] are still connected by landline, were good prerequisites for establishing a representative sample of participants. A migration background had 22.2% of the participants which is equivalent to the proportion in the population of NRW (23.1% in 2009). A pretest was conducted. The institute performing the CATI reported no problems with the understanding of the questions. Probing sentences were given in the survey to support the interviewers in explaining the definition of telemedical devices and to guarantee standardized explanations.

The survey included a high proportion of well-educated participants (*Abitur*: 32.8%). The majority of participants reported to be in good health (75.2%). These characteristics might have biased the knowledge of telemedical techniques and its purposes and options. In the Danish survey [25], participants with long-term higher education significantly more often approved the idea of a video consultation to get faster treatment (OR = 0.33; p < 0.01).

## Conclusion

The attitude towards telemedical devices strongly depends on age. The survey showed no difference in attitude toward the use of telemedical devices by rural vs. urban residency.

Many elderly fear the loss of direct contact to their physician. They feel a need for immediate feedback and explanation of measured values. For these, to know their vital status from measurement data on a display is not sufficient to feel comfortable in the case of illness. The skepticism in the elder age group was even stronger in the participants with chronic diseases.

In our population-based sample, less than two percent were experienced in using telemedical devices. Today's target patients for home telemonitoring have to be chosen carefully to ensure they can cope on their own despite they are less used to electronic communication. The fear of being left alone with the technique and unexplained measurement results needs to be compensated. For the patients their physicians are the interpreters of measurement values - even if patients are accustomed to a chronic state of their disease. An immediate trusted translation of data into "alert" or "all-clear" is needed. Additionally, people want to be sure of direct and personal medical help in severe acute conditions. More information on how this is managed in home telemonitoring will help to increase the willingness to use telemedical devices in general.

As a start these findings were disseminated into the model region for telemedicine [15] in NRW to improve consultations within the health care projects conducted in this region. Addressing patients' expectations and fears adequately may facilitate physician-patient-communication about telemedical supported therapies. A follow-up is planned to monitor changes in the attitude of the population in the model region, and to identify consultation strategies which are supportive for all German GPs, since home telemonitoring will spread.

## Abbreviations

CATI: Computer assisted telephone interviewing; COPD: Chronic obstructive pulmonary disease; CI: Confidence interval; GP: General practitioner; HS: High school qualification; ISCED-1997: International Standard Classification of Education 1997; LHS: Less than high school qualification; NRW: North Rhine-Westphalia; OR: Odds ratio; SD: Standard deviation; SUZ: Sozialwissenschaftliches umfragezentrum GmbH.

## Competing interests

The authors declare that they have no competing interests.

## Authors' contributions

CT has made substantial contributions to conception and design, analysis and interpretation of data; and drafted the manuscript. MM has made substantial contributions to conception and design, acquisition of data, and analysis of data. OM has been involved revising it critically for important intellectual content. All authors read and approved the final manuscript.

## Pre-publication history

The pre-publication history for this paper can be accessed here:

http://www.biomedcentral.com/1472-6963/12/95/prepub
